# Metabolomics Analysis of the Deterioration Mechanism and Storage Time Limit of Tender Coconut Water during Storage

**DOI:** 10.3390/foods9010046

**Published:** 2020-01-03

**Authors:** Yunwu Zhang, Wenxue Chen, Haiming Chen, Qiuping Zhong, Yonghuan Yun, Weijun Chen

**Affiliations:** College of Food Science and Engineering, Hainan University, Haikou 57022, China; z625967127@163.com (Y.Z.); hnchwx@163.com (W.C.); hmchen168@126.com (H.C.); hainufood88@163.com (Q.Z.); yunyonghuan@foxmail.com (Y.Y.)

**Keywords:** tender coconut water, differentially expressed metabolite, storage period, metabolic pathway, amino acid

## Abstract

Tender coconut water tastes sweet and is enjoyed by consumers, but its commercial development is restricted by an extremely short shelf life, which cannot be explained by existing research. UPLC-MS/MS-based metabolomics methods were used to identify and statistically analyze metabolites in coconut water under refrigerated storage. A multivariate statistical analysis method was used to analyze the UPLC-MS/MS datasets from 35 tender coconut water samples stored for 0–6 weeks. In addition, we identified other differentially expressed metabolites by selecting *p*-values and fold changes. Hierarchical cluster analysis and association analysis were performed with the differentially expressed metabolites. Metabolic pathways were analyzed using the KEGG database and the MetPA module of MetaboAnalyst. A total of 72 differentially expressed metabolites were identified in all groups. The OPLS-DA score chart showed that all samples were well grouped. Thirty-one metabolic pathways were enriched in the week 0–1 samples. The results showed that after a tender coconut is peeled, the maximum storage time at 4 °C is 1 week. Analysis of metabolic pathways related to coconut water storage using the KEGG and MetPA databases revealed that amino acid metabolism is one of the main causes of coconut water quality deterioration.

## 1. Introduction

The coconut (*Cocos nucifera* L.) originates from Palmae Cocos. Originally cultivated in Vietnam, Thailand, Myanmar, Malaysia, and the Philippines [1], coconuts are now also planted in Southern China, including Taiwan, Southern Yunnan, and other tropical areas. The fruit of the coconut is nearly round. A coconut has three layers of peel: Exocarp, mesocarp, and endocarp. The outermost layer, which is typically smooth with a greenish color, is called the exocarp. The next layer is the fibrous husk, or mesocarp, which ultimately surrounds the hard woody layer called the endocarp. Inside the endocarp are the solid endosperm (coconut meat) and liquid endosperm (coconut water). A tender coconut is one with a maturity of 6–9 months, and its water is directly consumed or processed into a variety of beverages. For half a century, extensive research has been conducted on the nutrition of coconut water, which contains 17 amino acids required by the human body [2]. The essential amino acids in coconut water are complete and nutrient-rich, and some are even more nutrient-dense than those in milk [3]. Moreover, coconut water has numerous medicinal properties, such as detoxification, antibacterial, anti-inflammatory, rejuvenation, digestion, and diuretic properties. Coconut water also has a therapeutic effect on gastric dysfunction, dysentery and child malnutrition and offers some control over hypertension, kidney stones, and urethral stones [4,5,6]. Moreover, coconut water affords protection against the induction of myocardial infarctions [7]. As a natural drink with high nutritional value, coconut water can replenish lost body fluids and alleviate electrolyte imbalance; in emergencies, coconut water can be injected intravenously into patients for hydration [8]. In addition, coconut water can be used as a plant tissue culture medium [9,10,11] because it has a variety of amino acids, cytokinins, auxin, and other nutrients [12].

For a large number of coconut farmers, the growing popularity of coconut water as a healthy drink may be very positive. However, research shows that tender coconuts can be stored for less than a month only under refrigerated conditions [13]. Fresh coconut water is sterile, so there are other reasons for its deterioration. Flavor compounds in tender coconut water may be formed from the degradation of fatty acids, which is probably caused by oxidative metabolism involving mechanisms such as p-oxidation and lipoxygenase (LOX) pathways [14]. During storage, the levels of nonanal and octanal, which could indicate the generation of an off-flavor, increase significantly in coconut water from commercially trimmed tender coconut. Furthermore, tender coconut stored at normal temperature exhibits obvious exocarp shriveling [15], continually increasing weight loss and the beginnings of exocarp browning and discoloration [16]. The high transportation costs and extremely short storage periods have restricted the development of tender coconut as a commercial product.

Tender coconut water undergoes a series of biological and chemical changes during harvesting. To date, research that uses metabolomics to study the changes in tender coconut water has been rare. Metabolomics is a research method similar to genomics and proteomics and is widely used to analyze changes in the quality of plants. Plant metabolomics mainly concerns metabolic analysis and metabolite fingerprinting of single plants, in which the metabolites in plant organs and tissues are qualitatively and quantitatively analyzed. In addition, metabolomics is used to compare and identify plants of the same species but different genotypes and to study the interaction between plants and herbivores, including the effects of plant defense metabolites on herbivore genotypes and resistance [17,18,19,20,21,22,23,24,25,26]. In this work, metabolomics was used to analyze the quality and metabolic changes in tender coconut during storage after harvest, and the important reasons for the deterioration of coconut were revealed, thus providing a theoretical basis for the preservation of fresh coconut after harvest.

## 2. Materials and Methods

### 2.1. Chemicals and Reagents

LC-MS-grade methanol was obtained from WOKAI. LC-MS-grade acetonitrile, 2-chlorophenylalanine, formic acid, and ammonium formate were obtained from Aladdin. Double-distilled water (ddH2O) was used.

### 2.2. Sample Collection

Hainan-native high-growing coconut was used as an example to study the growth characteristics of tender coconut. All coconuts were grown to 8 months old and had uniform color, with no surface damage or insect-borne infections. After the exocarp and mesocarp were removed, the coconuts were stored in a refrigerator at 4 °C. To eliminate any biological differences in the samples, the coconut water of 15 coconuts was taken every week, and the coconut water of every three coconuts was mixed into one sample, totaling 5 parallel samples. The coconut water samples were stored for 0 to 6 weeks, collected (coconut water collected in weeks 0–6 was the first to seventh groups of samples), packed in aluminum foil bags, frozen in liquid nitrogen and immediately placed in a freezer at −79 °C.

### 2.3. Determination of Physical and Chemical Indexes

The electroconductibility of tender coconut water was measured by an m-t-fe30 LCD-D instrument, the sugar content was measured by a Pal-2 hand-held saccharimeter, and the acidity and alkalinity were measured by an m-t-fe20 acidity-alkalinity meter.

### 2.4. Preparing Samples for UPLC-MS/MS Analysis

**Figure 1:**
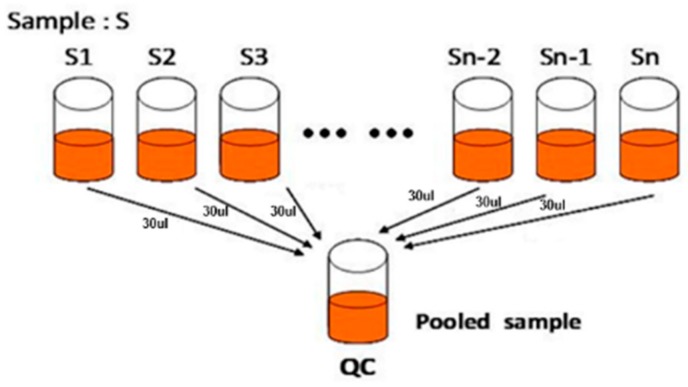
Preparation of the QC sample. Note: S1, S2, Sn-1, Sn, sample; QC, pooled samples for quality control.

All samples were thawed at 4 °C. Next, 100 mg of the sample was transferred to a 2 mL centrifuge tube containing 0.3 mL of ethanol, ultrasonicated for 30 min at 25 °C and centrifuged at 12,000 rpm for 10 min. The supernatant was filtered through a 0.22 µm membrane, and the obtained filtrate was analyzed by UPLC-MS/MS. Thirty microliters of filtrate was obtained from each supernatant and mixed to make the QC sample (Figure 1). Quality control samples were used to monitor the deviation in the analysis results of pooled sample mixtures and to compare this deviation with the error caused by the analyzer itself. The remaining samples were tested by UPLC-MS/MS [27,28].

### 2.5. Metabolomics Analysis Based on UPLC-MS/MS

A Thermo Ultimate 3000 system was used with an ACQUITY UPLC HSS T3 (2.1 × 150 mm, 1.7 µm) column and the following parameters: autosampler temperature of 8 °C, flow rate of 250 μL/min, column oven temperature of 38 °C, and sample size of 2 μL. Gradient elution was performed in positive ion mode with a mobile phase consisting of 0.1% formic acid in water (A) and 0.1% formic acid in acetonitrile (B) and in negative ion mode with a mobile phase consisting of 5 mM ammonium formate in water (C) and acetonitrile (D). The gradient elution procedure was as follows: 0–1 min, 2% B/D; 1–9 min, 2%–48% B/D; 9–12 min, 48%–98% B/D; 12–13.5 min, 98% B/D; 13.5–14 min, 98%–2% B/D; and 14–17 min, 2% B/D [29].

MS analysis was performed on a Thermo Q Exactive Focus with electrospray ionization (ESI) system with a positive ion spray voltage of 3.80 kV and a negative ion spray voltage of 2.50 kV. The capillary temperature was 325 °C, the resolution was 70,000, and the scanning range was 81–1000. Data dependent acquisition (DDA) MS/MS experiments were performed with HCD scan. The collision voltage was 30 eV. Dynamic exclusion was used to remove unnecessary MS/MS information [29].

### 2.6. Data Handling and Multivariate Statistical Analysis

The raw data (Supplementary Materials) obtained were translated into mzXML format by ProteoWizard [30]. Peak recognition, peak filtering, and peak alignment were performed using the edited R XCMS package [30,31]. The data were exported to Excel tables for calculation. Because data of different orders of magnitude were to be compared, the data were subjected to batch normalization of the peak area.

Due to the multidimensional nature of metabolomic data and the high correlation between certain variables, traditional univariate analysis could not be run quickly, fully and accurately to mine potentially useful information from the data. Therefore, when analyzing metabolomic data, it is important to use chemometrics and multivariate statistics for dimension reduction and classification of the collected multidimensional data to extract the most useful information. In this experiment, the data were subjected to autoscaling and mean-centering and were scaled to unit variance (UV) before multivariate statistical analysis to obtain more reliable and intuitive results. The multivariate statistical analysis (R language ropes package [32]) methods used were PCA, PLS-DA, and OPLS-DA.

## 3. Results

### 3.1. Physical and Chemical Indicators of Change

**Figure 2:**
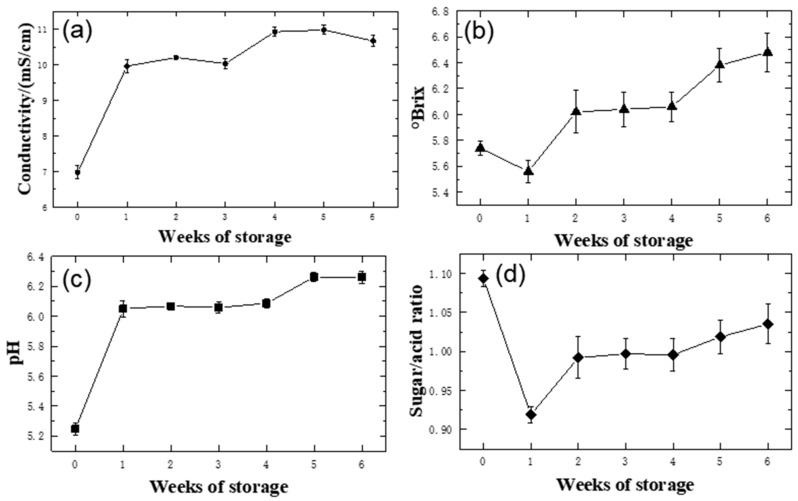
Changes in the conductivity (**a**), °Brix (**b**), pH (**c**) and sugar-to-acid ratio (**d**) of tender coconut water during storage.

The physical and chemical indicators corresponding to the storage time are shown in Figure 2. During weeks 0–1, the conductivity of the samples changed significantly (Figure 2a). However, there was no significant change in the conductivity of the samples from the first week of storage to the end (week 6). The °Brix of the samples decreased from 5.74 to 5.56 in weeks 0–1 but increased to 6.48 from the first week until the end (Figure 2b), reflecting drastic fluctuations during storage. The decrease in °Brix in weeks 0–1 may be due to high metabolism, while the continuous increase in °Brix in weeks 1–6 may be due to the decrease in metabolic activity and evaporation of coconut water during storage (a coconut loses approximately 10 g of water a week). In weeks 0–1, the pH of the samples quickly rose from 5.25 to 6.05. However, there was no significant change in sample pH from the first week to the sixth week of storage (Figure 2c). The increase in pH may have been due to organic acid consumption processes, such as the tricarboxylic acid cycle, gluconeogenesis, zymosis and amino acid interconversion [33]. This section is divided by subheadings and provides a concise and precise description of the experimental results, their interpretation and the experimental conclusions that can be drawn.

The ratio of sugar to acid (Figure 2d) is an important indicator that can affect the taste, quality and shelf life of food. The sugar-to-acid ratio of the samples dropped rapidly from 1.09 to 0.91 in, corresponding to the significant changes in pH and °Brix. However, from the first week to the end of storage (6 weeks), the pH of the samples increased slowly and steadily, while the °Brix increased steadily towards the end. From the first to the sixth week, the ratio of sugar to acid increased slowly, mainly because of the increase in °Brix. According to the sugar-to-acid ratio data, the flavor of tender coconut water changed significantly after one week of storage, which indicates that the storage period of the tender coconut water may not exceed one week.

### 3.2. Metabolic Profile Analysis of Tender Coconut Water during Storage

Positive ion mode (NT-pos) and negative ion mode (NT-neg) are two scanning modes of MS. After the sample is ionized by the ESI source, there will be ions with a positive charge (M + H, M + NH_4_, M + Na, etc.) and negative charge (M-H, M + Cl, M + CH_3_COO, etc.) at the same time. According to the differences in the physical and chemical properties of the substances, some metabolites will be positively charged, and some will be negatively charged. To obtain comprehensive metabonomics information, two modes are used simultaneously.

**Figure 3:**
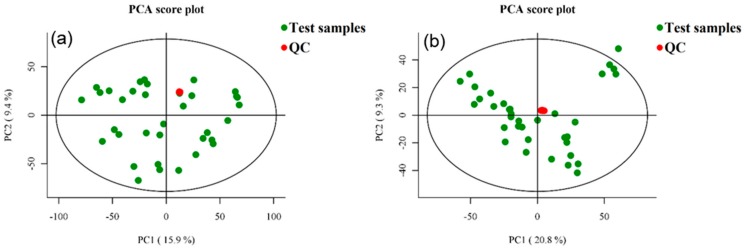
PCA score chart of QC samples under positive ion mode (NT-pos) (**a**) and negative ion mode (NT-neg) (**b**).

As shown in Figure 3, under the conditions of NT-pos and NT-neg, QC samples are intensively distributed with good repeatability, indicating that the system is stable.

**Figure 4:**
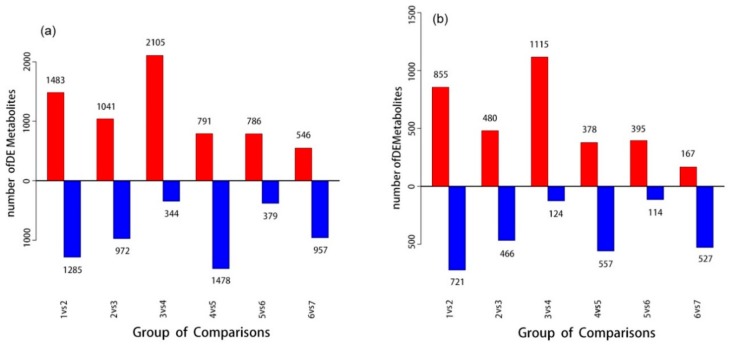
Up-regulated and down-regulated differentially expressed metabolites based on NT-pos (**a**) and NT-neg (**b**). The red color indicates up-regulated differentially expressed metabolites, and the blue color indicates down-regulated differentially expressed metabolites.

Metabolites are the products of metabolism in animals or plants. The up-regulated or down-regulated metabolites between the experimental group and the control group are called differentially expressed metabolites, which can identify abnormal metabolic changes and can also be discussed in combination with pathways related to differentially expressed metabolites. The differentially expressed metabolites are displayed in Figure 4a,b. In this study, metabolites were screened for differential expression, and the relevant conditions are as follows:

(1) *p*-value ≤ 0.05 and VIP ≥ 1 [34]
(2) One-way ANOVA *p*-value ≤ 0.05 [35]

*p*-value: Student’s t test, *p*-value ≤ 0.05 indicates a statistically significant difference. VIP: Variable Importance in the Projection; indicates the importance of variables to the model. One-way ANOVA *p*-value: Comparison of the mean of single factors and multiple independent samples. Student’s t test was used between two groups, and one-way ANOVA was used between multiple groups.

**Figure 5:**
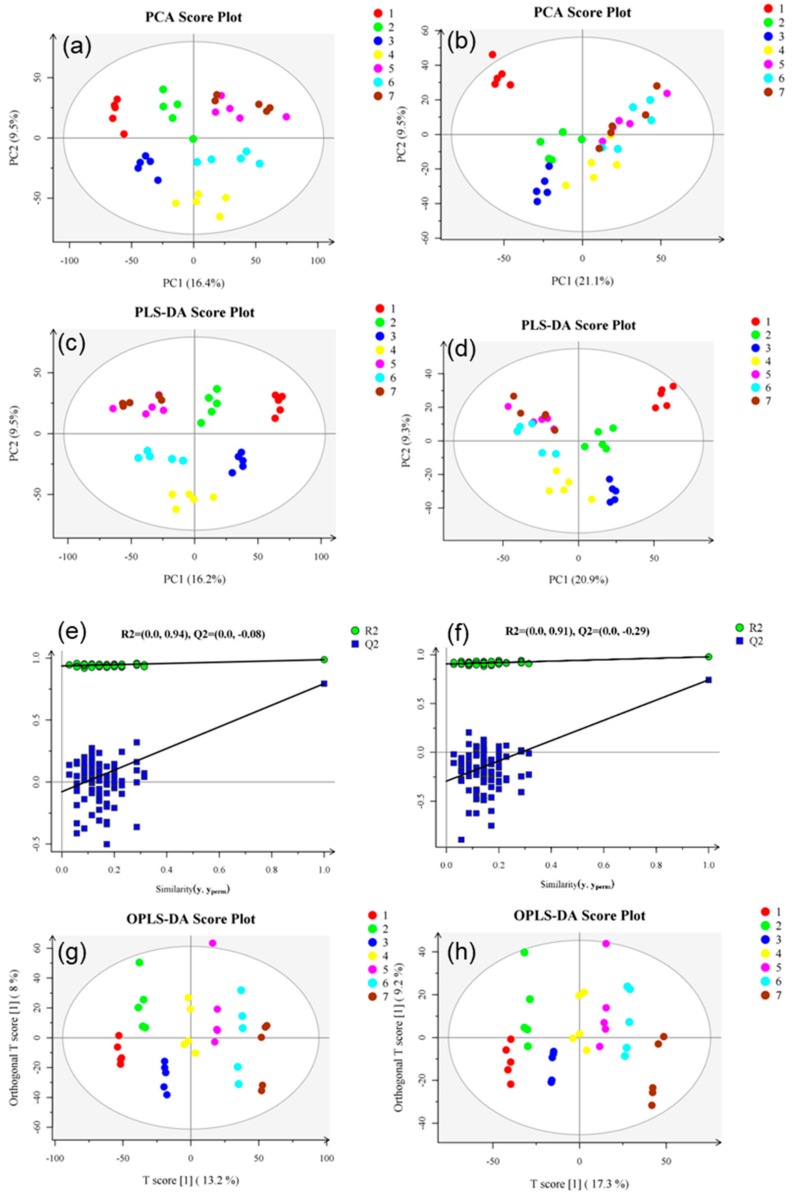
PCA score charts based on NT-pos (**a**) and NT-neg (**b**), PLS-DA score charts based on NT-pos (**c**) and NT-neg (**d**), PLS-DA permutation plot based on NT-pos (**e**) and NT-neg (**f**), and OPLS-DA score charts based on NT-pos (**g**) and NT-neg (**h**). R2, the interpretability of the model; Q2, the predictability of the model. Samples from 7 different groups are represented by 7 different colors, and each group has 5 biological replicates.

In this study, condition 1 + 2 was used for metabolite screening, Student’s t test was used between two groups, and one-way ANOVA was used between multiple groups. FDR was used to correct the *p*-value and control the final analysis results using the Benjamin–Hochberg (BH) [36] method. Positive ion mode was used to obtain 2923 differentially expressed metabolites, and negative ion mode was used to obtain 1695 differentially expressed metabolites. Figure 2 shows the statistical results of differentially expressed metabolites to be identified. PCA, PLS-DA, and OPLS-DA were analyzed in all groups, and a PCA score chart was drawn (Figure 5a,b). Under NT-pos conditions, the samples were all in the 95% confidence interval, the number of separation weeks was not enough, and the samples in groups 5 and 7 had a small overlap. Under NT-neg conditions, the number of separation weeks was very poor, most of the samples had 95% confidence intervals, except for one dot in group 1. PLS-DA showed some improvement (Figure 5c,d). Under NT-pos conditions, all samples were well differentiated, while under NT-neg conditions, the samples of groups 5, 6, and 7 overlapped. The OPLS-DA score showed that all samples were well differentiated under NT-pos and NT-neg conditions (Figure 5g,h). Permutation plots (Figure 5e,f) were used for effectively assessing whether the current PLS-DA model was over-fitting. Any one of the following criteria needed to be satisfied: (1) All Q2 points were lower than the rightmost original Q2 point; (2) the regression line of the Q2 point was less than or equal to 0 at the intersection of the ordinate. As shown, all blue Q2 points under NT-pos and NT-neg conditions were below the rightmost original blue Q2 point. Furthermore, the OPLS-DA score chart shows that all samples were well grouped. Compared with PLS-DA, OPLS-DA can effectively reduce the complexity of the model and enhance the interpretation ability of the model without reducing the prediction ability of the model to check the differences between groups to the greatest extent.

### 3.3. Metabolomics Analysis of Tender Coconut Water

**Figure 6:**
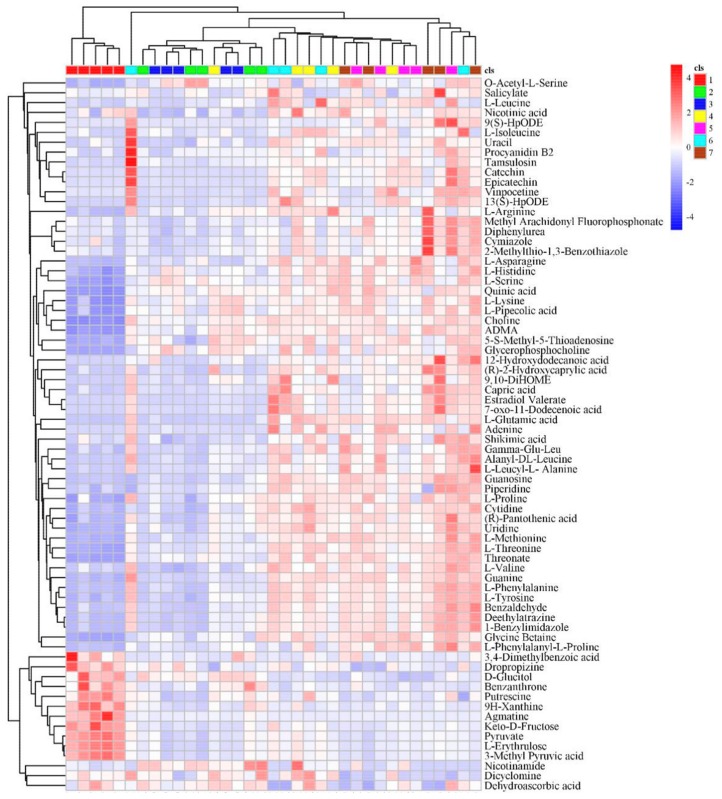
Heat map for the differentially expressed metabolites. The relative content in the figure is displayed by the color difference, where the columns represent the samples and the rows represent the metabolites. Samples from 7 different groups are represented by 7 different colors, and each group has 5 biological replicates.

To identify the metabolites, their exact molecular weight was confirmed (molecular weight error < 20 ppm). The fragment information (including the mass nuclear ratio, retention time and peak area of the identified metabolite) obtained according to the MS/MS mode was matched in the Human Metabolome Database (HMDB) (Metlin, MassBank, Lipid Maps, mzclound) and a self-built standards database to obtain accurate metabolite information. Finally, a total of 112 differentially expressed metabolites were matched (Table A1). Then, hierarchical clustering analysis was performed on each group of differentially expressed metabolites (Figure 6). At the bottom of the tree, each cluster extends a vertical line and then is aggregated by a horizontal line; each horizontal line is a category. The horizontal lines continue to aggregate from the bottom to the top. The more horizontal lines aggregate, the more concentrated the categories are. The top horizontal line of the tree divides the samples into two categories. When the distribution pattern of the two categories was observed, all samples in the first group (all of week 0) were distributed in the first category, while all others (week 1–6) were distributed in the second category. This finding indicates that the first and second groups of samples have the largest difference in metabonomics.

Z-score is calculated based on the relative content of metabolites [37,38], which is used to measure the relative content of metabolites at the same level, and the formula is:

z = (x − μ)/σ(1)

where x represents a specific fraction. μ represents average, while σ represents standard deviation.

**Figure 7:**
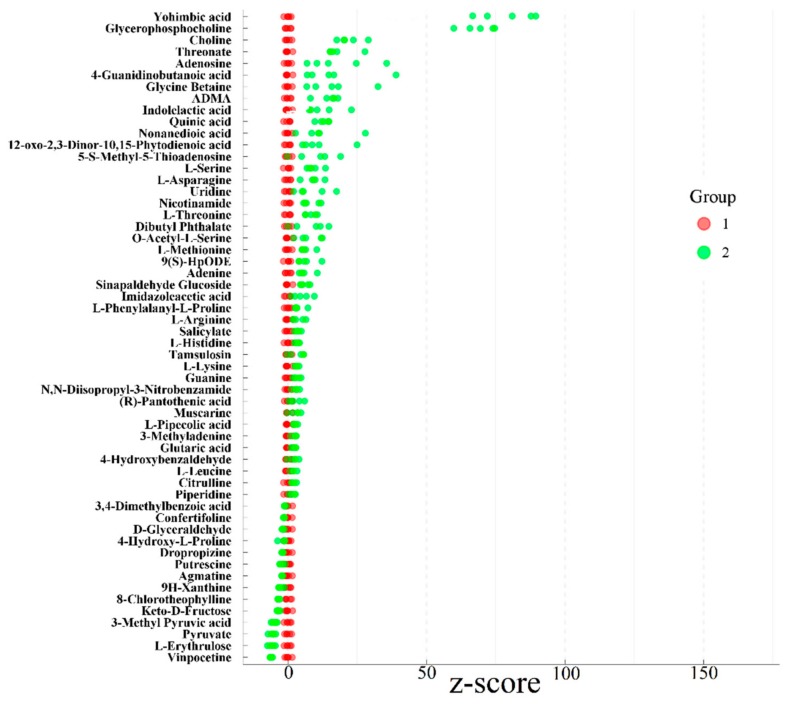
z-score chart of differentially expressed metabolites in the first and second group.

The statistical analysis of z-score is as shown in Figure 7. The z-score of the second group was calculated, while taking the first group as the control group. The metabolite is downregulated when the z-score is negative, and the metabolite is upregulated when the z-score is positive. The z-score chart shows that there are 52 upregulated differentially expressed metabolites and 14 downregulated differentially expressed metabolites in the first and second group. Yohimbic acid was thus identified as the most influential upregulated metabolite and vinpocetine was the most influential downregulated metabolite between groups 1 and 2.

**Figure 8:**
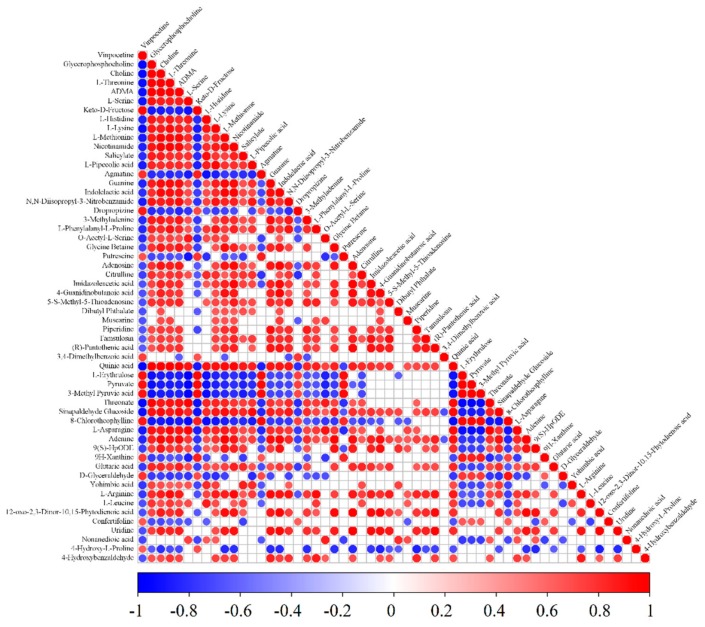
Correlation matrices of the first and second groups of samples of differentially expressed metabolites. The red color represents positive correlation, and the blue color represents negative correlation.

Differentially expressed metabolite correlation analysis was used to study the consistency of the trends among metabolites [39,40]. The correlations between individual metabolites were analyzed by calculating the Pearson correlation coefficients or the Spearman rank correlation coefficients for all metabolite pairs. Metabolite correlation often reveals the synergy of changes between metabolites: Positive correlation reflects the same trend for metabolites, and negative correlation reflects a different trend. This analysis was used to investigate the relationship between 56 major differentially expressed metabolites (Table A2) in all samples of the first and second groups. The correlation matrices of the differentially expressed metabolites are shown in Figure 8. In the figure, the blue dot indicates a negative correlation, and the red dot indicates a positive correlation. The correlation coefficient is between −1 and 1, and the different color depths are used to represent different correlations. When there is a strong linear relationship between two metabolites, the correlation coefficient is either 1 or −1, reflecting either a positive or negative correlation, respectively. In addition, the cor.test () function in R was used for statistical analysis of the metabolite association results, and a *p*-value ≤ 0.05 was considered significant. As shown in Figure 8, there are more than 20 connections between 12 types of amino acids and other metabolites, among which L-lysine is connected with all the other 55 metabolites. L-threonine is connected with 51 metabolites, and L-methionine is connected with 45 metabolites. It has also been confirmed that amino acids play an important role in metabolism.

**Figure 9:**
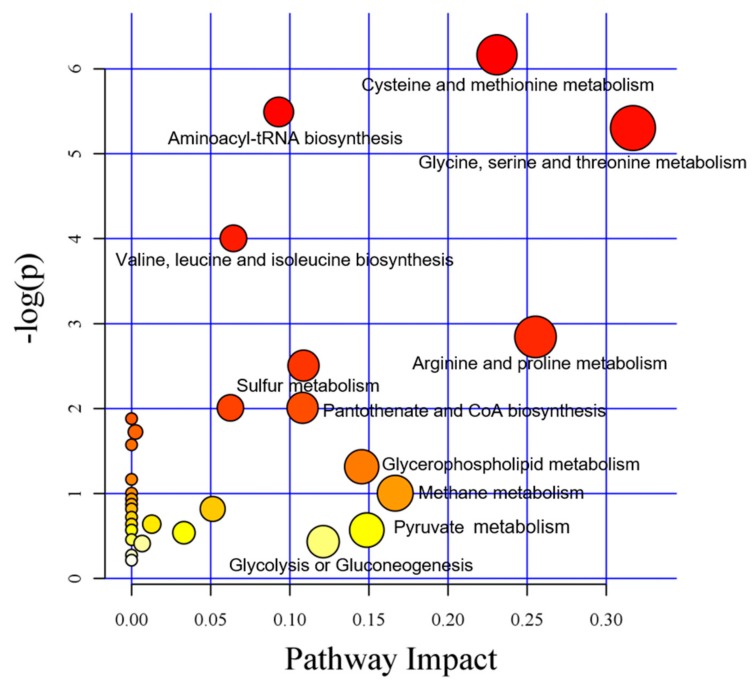
Analysis of metabolic pathways of the first and second groups of samples of differentially expressed metabolites. Each dot represents a metabolic pathway.

MetPA (www.metaboanalyst.ca) [41] is based mainly on the KEGG metabolic pathway. The MetPA database identifies metabolic pathways that may be disturbed by organisms through metabolic pathway concentration and topological analysis and is used to analyze the metabolic pathways of metabolites. The data analysis algorithm used is a hypergeometric test, and the topological structure of the pathway is relative betweenness centrality. Based on the metabolic pathway analysis performed using the KEGG pathway and MetPA database (Figure 9), the metabolic pathways enriched in 56 differentially expressed metabolites in the first and second groups were analyzed in this study. Thirty-one metabolic pathways were found, each represented by a dot. The larger the abscissa is, the larger the dot is, indicating that this metabolic pathway is more important to the metabolism of the sample. The larger the ordinate is, the darker the dot color is, indicating that this metabolic pathway is more enriched.

The main metabolic pathways are as follows. (1) In the metabolism of cysteine and methionine, cysteine is converted from serine (via acetylserine) by transfer of hydrogen sulfide and metabolized to pyruvate via multiple routes, and methionine is synthesized from aspartate [42,43]. (2) The metabolism of glycine, serine, and threonine results in 3-phospho-D-glycerate, which is an intermediate in glycolysis, produces serine and glycine, and reduces aspartate acid, which produces threonine in plants and bacteria [44]. (3) In the metabolism of arginine and proline, arginine is used to synthesize putrescine by arginase and ornithine decarboxylase, and glutamic acid is used to synthesize proline by delta-1-pyrroline-5-carboxylate synthetase, pyrroline-5-carboxylate reduce, glutamate 5-kinase and glutamate-5-semialdehydehydrogenase [45,46]. (4) Aminoacyl-tRNA biosynthesis increases amino acids [47,48]. (5) Valine, leucine and isoleucine biosynthesis increases amino acids through transamination of 3-phospho-D-glycerate and pyruvate consumption [44,49]. (6) Pantothenate and CoA biosynthesis produces 4’-phosphopantethein, which promotes the TCA cycle, β-oxidation, and fatty acid and polyketide biosynthesis pathways as an auxiliary factor.

The three most important metabolic pathways are amino acid pathways: (1) Glycine, serine and threonine metabolism; (2) arginine and proline metabolism; and (3) cysteine and methionine metabolism. Pyruvate, l-serine, 2-oxobutyric acid, l-leucine, putrescine, l-histidine, l-asparagine, l-threonine, pantothenic acid, *O*-acetyl-l-serine, l-lysine, l-arginine and l-methionine participate in at least two pathways, indicating that these substances are at the nodes of complex network pathways. Pyruvate is involved in 12 pathways, and serine is involved in 7 pathways, indicating that pyruvate and serine are the junction of each pathway. Moreover, pyruvate is closely related to amino acid metabolism. In previous studies, the changes in the quality of tender coconut water were attributed to aldehydes and lipids. In this study, amino acid metabolism was found to be another main cause of deterioration of tender coconut water. During the storage of tender coconut water, a variety of proteases play a key role in the metabolism of amino acids.

## 4. Conclusions

Samples taken at various time points during the storage of coconut water could be clearly divided into two categories by hierarchical clustering analysis: week 0 and weeks 1, 2, 3, 4, 5, and 6. The physical and chemical indicators showed significant metabolic differences between samples at week 0 and week 1. Thus, on the basis of metabolomics analysis, after tender coconut is peeled, the maximum storage time at 4 °C is week.

Metabonomics provides a direction for the study of physiological and biochemical deterioration of tender coconut during storage. After differentially expressed metabolites produced during coconut water storage were screened, KEGG and MetPA analysis of the metabolic pathways relevant to coconut water storage was performed and revealed that amino acid metabolism is one of the main causes of the deterioration of coconut water quality. These results provide a good theoretical basis for the preservation of tender coconut water.

## Supplementary Materials

The following are available online at https://www.mdpi.com/2304-8158/9/1/46/s1.

## Appendix A

**Table A1:** Differentially expressed metabolites in all groups.

Name ^1^	m/z ^2^	rt ^3^	ppm ^4^	Exact Mass ^5^	Formula	KEGG ^6^	Posneg ^7^
Piperidine	86.097103	130.617	8	85.1475	C_5_H_11_N	C01746	pos
Pyruvate	87.007167	95.2173	19	88.06206	C_3_H_4_O_3_	C00022	neg
D-Glyceraldehyde	89.02315	23.7384	8	90.07794	C_3_H_6_O_3_	C00577	neg
Acetoin	89.060419	75.4608	9	88.10512	C_4_H_8_O_2_	C00466	pos
Putrescine	89.107731	75.8816	10	88.1515	C_4_H_12_N_2_	C00134	pos
3-Methyl Pyruvic Acid	101.02312	94.7351	13	102.0886	C_4_H_6_O_3_	C00109	neg
Gamma-Aminobutyric Acid	104.07055	120.134	1	103.1198	C_4_H_9_NO_2_	C00334	pos
Choline	104.10692	90.8299	2	104.1708	C_5_H_14_NO	C00114	pos
l-Serine	106.05005	87.5235	2	105.09262	C_3_H_7_NO_3_	C00065	pos
Diethanolamine	106.08649	87.4857	2	105.13568	C_4_H_11_NO_2_	C06772	pos
Benzaldehyde	107.0494	353.073	3	106.1219	C_7_H_6_O	C00193	pos
Uracil	111.01883	122.908	11	112.08684	C_4_H_4_N_2_O_2_	C00106	neg
l-Proline	116.07017	101.442	0	115.1305	C_5_H_9_NO_2_	C00148	pos
l-Valine	116.0707	112.712	9	117.14638	C_5_H_11_NO_2_	C00183	neg
Glycine Betaine	118.08633	94.5577	2	117.14638	C_5_H_11_NO_2_	C00719	pos
l-Erythrulose	119.03418	94.3132	7	120.10392	C_4_H_8_O_4_	C02045	neg
l-Threonine	120.0656	92.1067	2	119.1192	C_4_H_9_NO_3_	C00188	pos
4-Hydroxybenzaldehyde	121.02785	536.412	14	122.12134	C_7_H_6_O_2_	C00633	neg
Nicotinic acid	122.02354	121.987	4	123.10944	C_6_H_5_NO_2_	C00253	neg
Nicotinamide	123.05547	116.543	0	122.12472	C_6_H_6_N_2_O	C00153	pos
Imidazoleacetic Acid	127.05018	101.293	0	126.114	C_5_H_6_N_2_O_2_	C02835	pos
4-Hydroxy-l-Proline	129.97455	135.595	16	131.1299	C_5_H_9_NO_3_	C01015	neg
5-Oxo-l-Proline	130.04944	195.121	2	129.114	C_5_H_7_NO_3_	C01879	pos
l-Pipecolic Acid	130.086	80.1096	1	129.157	C_6_H_11_NO_2_	C00408	pos
l-Leucine	130.08668	196.186	14	131.17296	C_6_H_13_NO_2_	C00123	neg
Glutaric Acid	131.03365	78.4799	13	132.11462	C_5_H_8_O_4_	C00489	neg
l-Asparagine	131.04501	88.3785	9	132.118	C_4_H_8_N_2_O_3_	C00152	neg
Agmatine	131.12911	79.1293	0	130.19162	C_5_H_14_N_4_	C00179	pos
l-Isoleucine	132.10067	271.563	7	131.17296	C_6_H_13_NO_2_	C00407	pos
Adenine	134.04598	219.269	9	135.1269	C_5_H_5_N_5_	C00147	neg
Threonate	135.02871	84.5419	9	136.10332	C_4_H_8_O_5_	C01620	neg
p-Salicylic acid	137.02327	115.089	8	138.12074	C_7_H_6_O_3_	C00156	neg
Salicylate	139.11118	706.72	4	138.122	C_7_H_6_O_3_	C00805	pos
4-Guanidinobutanoic Acid	146.09205	121.961	0	145.1597	C_5_H_11_N_3_O_2_	C01035	pos
l-Glutamine	147.07625	90.527	2	146.14458	C_5_H_10_N_2_O_3_	C00064	pos
l-Lysine	147.11259	80.1761	1	146.18764	C_6_H_14_N_2_O_2_	C00047	pos
O-Acetyl-L-Serine	148.06013	93.1268	6	147.1293	C_5_H_9_NO_4_	C00979	pos
l-Glutamic Acid	148.06014	102.515	3	147.1293	C_5_H_9_NO_4_	C00025	pos
l-Methionine	150.05874	144.366	0	149.21238	C_5_H_11_NO_2_S	C00073	pos
3-Methyladenine	150.07725	120	6	149.15348	C_6_H_7_N_5_	C00913	pos
9H-Xanthine	151.02495	204.337	9	152.11102	C_5_H_4_N_4_O_2_	C00385	neg
3,4-Dimethylbenzoic acid	151.07466	356.095	5	150.1745	C_9_H_10_O_2_		pos
Guanine	152.05492	121.98	12	151.126	C_5_H_5_N_5_O	C00242	pos
l-Histidine	156.07667	86.0491	2	155.15468	C_6_H_9_N_3_O_2_	C00135	pos
1-Benzylimidazole	159.09162	405.939	1	158.084398	C_10_H_10_N_2_		pos
(R)-2-Hydroxycaprylic Acid	159.10166	390.322	7	160.2108	C_8_H_16_O_3_		neg
l-Phenylalanine	164.07031	330.364	12	165.18918	C_9_H_11_NO_2_	C00079	neg
Capric Acid	171.13816	806.583	5	172.265	C_10_H_20_O_2_	C01571	neg
Dehydroascorbic Acid	173.00812	112.035	6	174.10824	C_6_H_6_O_6_	C05422	neg
Shikimic Acid	173.04464	84.3461	5	174.1513	C_7_H_10_O_5_	C00493	neg
l-Arginine	173.10357	96.2243	9	174.201	C_6_H_14_N_4_O_2_	C00062	neg
Muscarine	174.14873	335.354	1	173.141579	C_9_H_19_NO_2_		pos
Citrulline	176.1028	94.1013	1	175.18584	C_6_H_13_N_3_O_3_	C00327	pos
l-Tyrosine	180.06494	122.902	9	181.18858	C_9_H_11_NO_3_	C00082	neg
Keto-D-Fructose	180.08643	96.8	7	180.15588	C_6_H_12_O_6_	C10906	pos
2-Methylthio-1,3-Benzothiazole	182.00909	815.127	1	181.278	C_8_H_7_NS_2_	C10910	pos
Triethyl Phosphate	183.07879	612.399	4	182.15466	C_6_H_15_O_4_P		pos
D-Glucitol	183.08768	93.504	8	182.17176	C_6_H_14_O_6_	C00794	pos
Nonanedioic Acid	187.09744	302.361	1	188.22094	C_9_H_16_O_4_	C08261	neg
3-Hydroxycapric Acid	187.13294	586.019	6	188.264	C_10_H_20_O_3_		neg
Deethylatrazine	188.07031	405.939	3	187.0625	C_6_H_10_ClN_5_	C06559	pos
Quinic Acid	191.05511	84.3797	5	192.16658	C_7_H_12_O_6_	C00296	neg
*N,N*-Diethyl-M-Toluamide	192.13757	755.649	4	191.2695	C_12_H_17_NO	C10935	pos
Butylparaben	193.08626	761.377	4	194.227	C_11_H_14_O_3_	D01420	neg
l-Leucyl-l-Alanine	201.1237	125.201	4	202.2508	C_9_H_18_N_2_O_3_		neg
Alanyl-DL-Leucine	203.13918	182.305	1	202.131742	C_9_H_18_N_2_O_3_		pos
ADMA	203.15006	101.974	1	202.25428	C_8_H_18_N_4_O_2_	C03626	pos
Indolelactic Acid	206.08104	486.255	1	205.073893	C_11_H_11_NO_3_	C02043	pos
7-Oxo-11-Dodecenoic Acid	211.13328	598.679	3	212.141237	C_12_H_20_O_3_		neg
8-Chlorotheophylline	213.01457	486.288	18	214.025751	C_7_H_7_ClN_4_O_2_		neg
Diphenylurea	213.10194	713.78	1	212.248	C_13_H_12_N_2_O		pos
Octhilinone	214.12574	808.599	1	213.34	C_11_H_19_NOS	C18752	pos
Tetradecylamine	214.25247	777.08	2	213.245649	C_14_H_31_N		pos
12-Hydroxydodecanoic Acid	215.16486	616.949	2	216.3172	C_12_H_24_O_3_	C08317	neg
Cymiazole	219.09484	780.674	1	218.087769	C_12_H_14_N_2_S		pos
(R)-Pantothenic Acid	220.11782	366.856	1	219.23502	C_9_H_17_NO_5_	C00864	pos
Benzanthrone	231.08369	121.982	14	230.073165	C_17_H_10_O		pos
Confertifoline	233.15282	691.733	8	234.335	C_15_H_22_O_2_	C09376	neg
Dropropizine	237.15702	504.911	12	236.311	C_13_H_20_N_2_O_2_		pos
l-Cystine	239.12849	758.968	3	240.30256	C_6_H_12_N_2_O_4_S_2_	C00491	neg
Uridine	243.06232	123.067	0	244.20146	C_9_H_12_N_2_O_6_	C00299	neg
Cytidine	244.09223	121.485	1	243.21674	C_9_H_13_N_3_O_5_	C00475	pos
*N,N*-Diisopropyl-3-Nitrobenzamide	251.13594	144.97	12	250.131743	C_13_H_18_N_2_O_3_		pos
Glycerophosphocholine	258.10912	92.1093	4	257.2213	C_8_H_20_NO_6_P	C00670	pos
Gamma-Glu-Leu	261.14469	317.015	1	260.2869	C_11_H_20_N_2_O_5_		pos
l-Phenylalanyl-l-Proline	263.13858	444.483	2	262.3043	C_14_H_18_N_2_O_3_		pos
12-oxo-2,3-Dinor-10,15-Phytodienoic Acid	263.16518	831.168	0	264.36	C_16_H_24_O_3_		neg
Adenosine	268.10341	309.894	0	267.24152	C_10_H_13_N_5_O_4_	C00212	pos
16-Hydroxy Hexadecanoic Acid	271.22753	833.483	1	272.4235	C_16_H_32_O_3_	C18218	neg
Triethylcitrate	277.12763	716.368	2	276.283	C_12_H_20_O_7_	D06228	pos
Dibutyl Phthalate	279.16034	797.891	4	278.3435	C_16_H_22_O_4_	C14214	pos
Guanosine	284.0984	316.055	4	283.24092	C_10_H_13_N_5_O_5_	C00387	pos
Epicatechin	289.0712	462.296	2	290.2681	C_15_H_14_O_6_	C09727	neg
Catechin	291.08533	462.105	5	290.2681	C_15_H_14_O_6_	C06562	pos
Terbinafine	292.21163	650.276	19	291.4299	C_21_H_25_N	C08079	pos
5-S-Methyl-5-Thioadenosine	298.09601	392.891	0	297.3347	C_11_H_15_N_5_O_3_S	C00170	pos
TMS	301.14395	420.822	2	300.136159	C_18_H_20_O_4_		pos
Dicyclomine	310.27177	755.879	8	309.4867	C_19_H_35_NO_2_	C06951	pos
Triptophenolide	311.16832	790.124	10	312.172545	C_20_H_24_O_3_		neg
13(S)-HpODE	311.22274	743.286	0	312.4443	C_18_H_32_O_4_	C04717	neg
9(S)-HpODE	311.22294	775.306	0	312.4443	C_18_H_32_O_4_	C14827	neg
9,10-DiHOME	313.23891	724.13	2	314.4602	C_18_H_34_O_4_	C14828	neg
Acitretin	325.18414	810.001	10	326.42934	C_21_H_26_O_3_	D02754	neg
Quinine	325.191	652.018	0	324.4168	C_20_H_24_N_2_O_2_	C06526	pos
Yohimbic Acid	339.1648	831.469	19	340.41624	C_20_H_24_N_2_O_3_		neg
Vinpocetine	351.21345	714.679	19	350.455	C_22_H_26_N_2_O_2_		pos
Estradiol Valerate	355.22845	599.118	2	356.499	C_23_H_32_O_3_	C12859	neg
Sinapaldehyde Glucoside	369.11997	96.3153	2	370.126376	C_17_H_22_O_9_		neg
Methyl Arachidonyl Fluorophosphonate	371.24766	763.893	9	370.243695	C_21_H_36_FO_2_P		pos
Gentian Violet	372.24226	790.875	3	371.236135	C_25_H_30_N_3_		pos
Tamsulosin	409.18158	464.483	6	408.512	C_20_H_28_N_2_O_5_S	C07124	pos
Procyanidin B2	579.1489	407.114	1	578.5202	C_30_H_26_O_12_	C17639	pos

^1^ Name: identification results; ^2^ m/z: mass nuclear ratio; ^3^ rt: retention time, s; ^4^ ppm: error between molecular weight and theoretical molecular weight, ppm; ^5^ exact mass: accurate molecular weight; ^6^ KEGG: KEGG compound number; ^7^ posneg: ionization mode, where pos is positive ion mode and neg is negative ion mode.

**Table A2:** Differentially expressed metabolites in all samples of the first and second groups.

Name ^1^	1 vs. 2_VIP ^2^	Fold Change_2/1	Log_2_(FC_2/1) ^3^	*p*-Value ^4^	FDR ^5^
Vinpocetine	1.869946	0.3202	−1.6429	0.0000013	0.000254
Glycerophosphocholine	1.856177	42.044	5.3938	0.0000037	0.000517
Choline	1.855588	6.291	2.6533	0.0000039	0.000531
l-Threonine	1.813788	1.6411	0.71469	0.0000320	0.002047
ADMA	1.808401	4.7429	2.2458	0.0000392	0.002287
l-Serine	1.790296	3.2158	1.6852	0.0000727	0.003252
Keto-D-Fructose	1.77673	0.20739	−2.2696	0.0001089	0.00426
l-Histidine	1.740644	2.6231	1.3913	0.0002689	0.007746
l-Lysine	1.739258	2.5496	1.3503	0.0002773	0.007874
l-Methionine	1.734536	4.0561	2.0201	0.0003074	0.00837
Nicotinamide	1.727199	5.4063	2.4346	0.0003589	0.009191
Salicylate	1.721537	1.1119	0.15298	0.0004026	0.010029
l-Pipecolic acid	1.648997	2.1215	1.0851	0.0013724	0.020324
Agmatine	1.629025	0.097098	−3.3644	0.0018093	0.02423
Guanine	1.62376	2.0952	1.0671	0.0019395	0.025193
Indolelactic acid	1.621818	4.6738	2.2246	0.0019892	0.025604
*N,N*-Diisopropyl-3-Nitrobenzamide	1.614857	2.2462	1.1675	0.0021749	0.027391
Dropropizine	1.576419	0.89283	−0.16354	0.0034327	0.035944
3-Methyladenine	1.569726	2.1357	1.0947	0.0036958	0.037589
l-Phenylalanyl-L-Proline	1.54751	2.9302	1.551	0.0046725	0.04372
*O*-Acetyl-l-Serine	1.534309	2.9405	1.5561	0.0053332	0.048166
Glycine Betaine	1.526096	4.1876	2.0661	0.0057765	0.050485
Putrescine	1.49701	0.56468	−0.8245	0.0075577	0.058673
Adenosine	1.492166	7.3509	2.8779	0.0078881	0.060157
Citrulline	1.484936	1.6943	0.76068	0.0084002	0.062645
Imidazoleacetic acid	1.414003	1.6133	0.69005	0.0147345	0.087407
4-Guanidinobutanoic acid	1.390837	12.812	3.6794	0.0173733	0.096289
5-S-Methyl-5-Thioadenosine	1.368643	4.2794	2.0974	0.0201933	0.105666
Dibutyl Phthalate	1.363868	3.1017	1.6331	0.0208389	0.107572
Muscarine	1.316996	3.677	1.8785	0.0279490	0.12836
Piperidine	1.275156	1.9234	0.94366	0.0355599	0.148035
Tamsulosin	1.271617	4.6915	2.23	0.0362616	0.149595
(R)-Pantothenic acid	1.224623	1.732	0.7924	0.0464829	0.171966
3,4-Dimethylbenzoic acid	1.222463	0.18966	−2.3985	0.0469943	0.173234
Quinic acid	1.780892	4.5158	2.175	0.0000016	0.000236
l-Erythrulose	1.739383	0.32058	−1.6412	0.0000213	0.001223
Pyruvate	1.736553	0.354	−1.4982	0.0000241	0.0013
3-Methyl Pyruvic acid	1.728988	0.32725	−1.6115	0.0000332	0.001502
Threonate	1.712791	3.4506	1.7868	0.0000607	0.002173
Sinapaldehyde Glucoside	1.701181	3.2492	1.7001	0.0000889	0.002691
8-Chlorotheophylline	1.700766	0.39072	−1.3558	0.0000901	0.0027
l-Asparagine	1.657314	5.2423	2.3902	0.0002870	0.006038
Adenine	1.585462	1.975	0.98184	0.0011264	0.015959
9(S)-HpODE	1.504033	2.4481	1.2917	0.0034110	0.032279
9H-Xanthine	1.489229	0.42715	−1.2272	0.0040398	0.03549
Glutaric acid	1.472775	3.4595	1.7906	0.0048316	0.039341
D-Glyceraldehyde	1.457553	0.20576	−2.281	0.0056575	0.042594
Yohimbic acid	1.447777	37.215	5.2178	0.0062382	0.045236
l-Arginine	1.428014	3.3292	1.7352	0.0075394	0.050283
l-Leucine	1.378902	2.4788	1.3096	0.0115875	0.063966
12-oxo-2,3-Dinor-10,15-Phytodienoic acid	1.360253	2.2232	1.1527	0.0134607	0.070293
Confertifoline	1.346373	0.28192	−1.8266	0.0149854	0.075265
Uridine	1.327309	3.6455	1.8661	0.0172704	0.082697
Nonanedioic acid	1.311257	9.5381	3.2537	0.0193733	0.088133
4-Hydroxy-l-Proline	1.256131	0.52658	−0.92528	0.0279380	0.109387
4-Hydroxybenzaldehyde	1.161319	2.5676	1.3604	0.0481468	0.151292

^1^ Name: identification results; ^2^ VIP: Variable Importance in the Projection; ^3^ log_2_ (FC): log_2_ value of fold change; ^4^ *p*-value: Student’s t test; ^5^ FDR: False Discovery Rate.
